# Where is VEGF in the body? A meta-analysis of VEGF distribution in cancer

**DOI:** 10.1038/sj.bjc.6603923

**Published:** 2007-10-02

**Authors:** C Kut, F Mac Gabhann, A S Popel

**Affiliations:** 1 Department of Biomedical Engineering, Johns Hopkins University School of Medicine, Baltimore, MD 21205, USA

**Keywords:** breast cancer, colorectal cancer, prostate cancer, serum, platelets, leukocytes

## Abstract

Vascular endothelial growth factor (VEGF) is a major target for the inhibition of tumour vascularisation and the treatment of human cancer. Many tumours produce large quantities of VEGF, and as a result, diagnosis and prognosis of cancer may be predicted by measuring changes in VEGF concentrations in blood. In blood, the VEGF may be located in the plasma, or in the blood-borne cells and formed elements, in particular, platelets and leukocytes. In this study, we collate the measurements of VEGF in platelets, leukocytes, plasma and serum for breast, prostate, colorectal and other cancers. In addition, we analysed the concentration of VEGF in tumour tissue itself, as well as for other tissues in the human body. Although the concentration of VEGF in tumours is high, the size of tumours is small compared to other tissues, in particular, skeletal muscle. Thus, the total quantity of VEGF in tumours and in blood is small compared to the quantity in muscles. This large reservoir of VEGF may have important implications for the treatment of cancer.

Angiogenesis, the development of new blood vessels from pre-existing vasculature, has important roles in growth and development, wound healing and tumorigenesis. The vascular endothelial growth factor (VEGF) family of proteins has a pivotal role in regulating tumour angiogenesis (Shibuya and Claesson-Welsh, 2006).

Vascular endothelial growth factor stimulates cell survival, migration and differentiation. It induces neovascularisation, and is required for the establishment of haematopoiesis; in malignant tumours, VEGF supports development of tumour vessels, which may lead to increased vascular permeability, and is shown to have a correlation with cancer prognosis and diagnosis (Folkman, 1995; Hormbrey *et al*, 2002).

A marked increase in VEGF levels has been observed in various types of cancer including anal carcinoma (Kusumanto *et al*, 2003), lymphoma (Salven *et al*, 1999a), lung cancer (Salgado *et al*, 1999; Yanagawa *et al*, 1999; Matsuyama *et al*, 2000; Kishiro *et al*, 2002), gastric carcinoma (Hyodo *et al*, 1998; Kraft *et al*, 1999; Yoshikawa *et al*, 2000), ovarian cancer (Hyodo *et al*, 1998; Tempfer *et al*, 1998; Kraft *et al*, 1999; Yoshikawa *et al*, 2000), renal cell carcinoma (Dosquet *et al*, 1997; Sato *et al*, 1999; Jacobsen *et al*, 2002; Ljungberg *et al*, 2003), brain tumour (Stockhammer *et al*, 2000), hepatocellular carcinoma (Poon *et al*, 2001), breast cancer (Bando *et al*, 2005; O’Riain *et al*, 2005), prostate cancer (Kaushal *et al*, 2005; Li *et al*, 2005) and colorectal cancer (Haraguchi *et al*, 2002; Karayiannakis *et al*, 2002; Werther *et al*, 2003). Of these, the last three are most extensively studied.

Both breast and prostate cancer are commonly diagnosed malignancies, and are among the top leading causes of death responsible for 15 and 10% of cancer deaths in women and men, respectively in 2005 (Uzzan *et al*, 2004; Jemal *et al*, 2005). Colorectal cancer is responsible for 10% of all cancer deaths in 2005 (Jemal *et al*, 2005). For all three cancer types, significant correlations between VEGF and the extent of tumour vascularisation, tumour stages and metastasis have been reported (Duque *et al*, 1999; Gasparini, 2000; Karayiannakis *et al*, 2002). In addition, it is reported that the status of oestrogen (ER) and progesterone (PgR) receptors may have direct correlation with VEGF level in breast cancer patients (Duque *et al*, 1999; Gasparini, 2000; Eccles, 2001; Karayiannakis *et al*, 2002). In recent years, VEGF has been considered a significant indicator of cancer, and blood VEGF levels are often used to estimate the degree of tumour development.

However, the debate is still on as to the origin and location of VEGF. Serum, plasma and whole blood have been commonly used to determine VEGF levels in the body, but it is not clear which measurement can provide the best prognostic information. Plasma is the free circulating, liquid component of blood, in which blood-formed elements are suspended. Serum is plasma with all coagulation factors removed, and is obtained by clotting the blood before centrifugation. Because coagulation results in the release of VEGF from platelets, serum VEGF concentration counts both plasma VEGF and platelet-held VEGF.

A number of studies report correlation between platelet counts and serum VEGF (Werther *et al*, 2002b; Caine *et al*, 2004), and higher serum VEGF levels per platelet in cancer (Salven *et al*, 1999a; Kusumanto *et al*, 2003). The importance of platelet-derived VEGF in cancer may be due to VEGF released upon thrombin activation by platelets, with VEGF inducing vascular permeability and in doing so further promoting coagulation (Mohle *et al*, 1997; Verheul and Pinedo, 1998). Other studies suggest that leukocytes are more important sources of VEGF in cancer patients. A similar mechanism concerning permeability and thrombin activation has been postulated to account for the leukocyte–VEGF interaction (Mohle *et al*, 1997; Salven *et al*, 1999a; Kusumanto *et al*, 2003). To determine platelet VEGF, values for platelet-rich plasma (PRP) and platelet-poor plasma (PPP) have been compared; for leukocytes, the values from a peripheral blood mononuclear cell suspension (PBMNC) have been obtained.

Studies on VEGF levels in urine (Bok *et al*, 2001), pleural effusion (Kraft *et al*, 1999; Thickett *et al*, 1999; Yanagawa *et al*, 1999; Kishiro *et al*, 2002; Toi *et al*, 2002), tumour cytosol (Obermair *et al*, 1997; Eppenberger *et al*, 1998; Baker *et al*, 2000; Broll *et al*, 2001; Foekens *et al*, 2001; Haraguchi *et al*, 2002; Toi *et al*, 2002; Manders *et al*, 2003; Desruisseau *et al*, 2004; Bando *et al*, 2005), tumour cyst fluid (Stockhammer *et al*, 2000) and other body fluids are available. However, most do not have a basis of comparison, as they report only the VEGF levels in cancer patients, but no control equivalent in healthy volunteers. It may be possible to compare some of these to values in healthy individuals from other studies.

The inhibition of VEGF expression and signalling in tumours is a promising therapeutic strategy. Tumour-induced angiogenesis is largely dependent on VEGF, and studies have demonstrated that anti-VEGF antibodies successfully inhibit both angiogenesis and tumour growth (Eatock *et al*, 2000). The first anti-VEGF drug, bevacizumab, was approved by Food and Drug Administration in 2004. In all phase trials, the drug was reported to be well-tolerated, and increased the response and survival rates of patients (Tortora *et al*, 2004).

In contrast to VEGF inhibition in tumours and other diseases, administration of additional VEGF could potentially treat disorders that result in restricted or limited blood supply. For example, it may be a therapeutic agent for chronic limb ischaemia, which is often caused by obstructive atherosclerosis and has a high mortality rate; other potential therapeutic applications include the treatment of coronary insufficiency and restenosis (Ferrara and Davis-Smyth, 1997).

Understanding the distribution of VEGF in human body is vital to the prognosis and treatment of cancer and other disorders. It is especially important for designing and understanding anti-VEGF therapeutics. However, important variations across studies, including methods of sample collection, patient selection, measurement units, statistical analysis and data interpretation, make it difficult to obtain a global view of VEGF distribution. To our knowledge, there has been no comprehensive review of the literature that elucidates the relative concentrations of VEGF in such body compartments as blood, normal tissues and organs, and tumour, nor one that gives detailed overview of VEGF levels across various cancer types. The present study aims to review previously reported VEGF levels and summarise the results in the form of VEGF localisation to various body compartments. An additional motivation and aim of the present study is to provide a solid basis for quantitative, systems biology studies of the VEGF system in health and disease (Mac Gabhann and Popel, 2006; Mac Gabhann *et al*, 2006).

## METHODS

### Publication selection

Meta-analysis was based on an electronic literature search through Pubmed and Google Scholar. Key words used included: vascular endothelial growth factor; VEGF; cancer; breast; prostate; colorectal; serum; plasma; platelets; and leukocytes. Papers were also found through the references and citations of all the relevant studies. To be included in our meta-analysis, papers had to be *in vivo* studies, included in the Pubmed database, deal with cancer patients and present quantitative VEGF data. Studies with aberrant data (over a 10-fold difference than those reported in other papers) were also excluded. Based on these criteria, 12 studies were excluded from our collection (Liu *et al*, 1999; Haggstrom *et al*, 2000; Bhujwalla *et al*, 2001; Feldman *et al*, 2001; George *et al*, 2001; Huss *et al*, 2001; Kelavkar *et al*, 2001; Calvo *et al*, 2002; Mabjeesh *et al*, 2003; Cianchi *et al*, 2004; Singh *et al*, 2004; May *et al*, 2005) (Supplementary Table S6).

### Information extraction

The following items were extracted from each paper: type of cancer, sample size, location of VEGF measured, VEGF isoform studied, methodology of data collection, VEGF diagnosis, statistical format, platelet/leukocyte count and the mean or median value for both healthy and cancer VEGF concentration. If the paper presented VEGF data in several categories and failed to report an overall cancer VEGF value, data estimation (indicated by an asterisk ^*^ in Supplementary Tables S1–S4, online supplement) was performed taking the average of data in all categories. Our analysis was performed by comparing results from individual papers. We did not amend the statistical analyses used in each paper.

### Vascular endothelial growth factor measurement methods used

Both plasma and serum have been commonly used to determine VEGF levels in the blood. From our reviewed literature, peripheral venous blood samples were drawn. To prepare plasma, blood samples were put in test tubes with an anticoagulant (either trisodium citrate or ethylenediaminetetraacetic acid). The test tubes were then left for 0–30 min before centrifugation, 1000–3000 × **g** at 4–21°C for 10–20 min. To prepare serum, blood samples were put in sterile silicone-coated tubes without additive, or serum separator/clot activator test tubes. Blood samples were then allowed to clot for 30–120 min before centrifugation, 1000–3000 × **g** at 4–21°C, for 7–15 min (one paper reported a centrifugation rate of 16 000 × **g**). Both plasma and serum samples were subsequently aliquoted and stored at −20 to −80°C before assay.

To obtain tumour cytosol measurements, tumour tissues obtained during surgery were immediately frozen in liquid nitrogen. To prepare the samples, the tissue samples were diluted in a buffer. The tissues were then either homogenised with Ultra Terrex, pulverised with microdismembrator or diced with a scalpel. The homogenised tissue samples were then centrifuged at 800–105 000 × **g**, for 15–60 min. The resulting supernatant (tumour cytosol) was then stored at −70 to −80°C until analysis.

Enzyme-linked immunosorbent assay (ELISA) was the adopted assay type for 74% of the studies. Among those using ELISA, 40% obtained the assay kit from R&D Systems, (Minneapolis, MN, USA). The remaining 60% used kits from 14 other different companies. Five other different assay types were also used, including quantitative sandwich enzyme immunoassay, enzyme immunoassay, chemiluminescence immunosorbent assay, human VEGF immunoassay quantigo kit and immunofluorometric assay.

### Data analysis

The weighted average and s.d. of VEGF concentrations were evaluated in all cancer studies for various compartment of the body (serum, plasma, whole blood and tumour cytosol). Our calculations were based on the following equations: 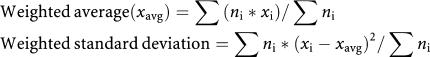


*n*_i:_ number of subjects reported in each paper; *x*_i_, VEGF level reported in each paper.

A two-sample, one-tailed Student's *t*-test was used. A probability of <0.05 was taken to be significant. The overall statistical meta-analysis is given in Table 1. In our meta-analyses, units were reported in pg ml^−1^, pg mg^−1^ protein, pg 10^−6^ cells, 10^6^ cells ml^−1^. Unit conversion was performed whenever necessary. Concentrations are rounded to whole numbers.

## RESULTS AND DISCUSSION

### Study selection

Our literature search identified 64 references containing quantitative information to be included in this analysis, including 19 breast cancer, 13 prostate cancer, 13 colorectal cancer and 19 other cancer studies. Details for each of these studies are given in Supplementary Table S1 for breast cancer (Yamamoto *et al*, 1996; Obermair *et al*, 1997; Salven *et al*, 1997, 1999b; Verheul *et al*, 1997; Eppenberger *et al*, 1998; Adams *et al*, 2000; Foekens *et al*, 2001; Heer *et al*, 2001; Colleoni *et al*, 2002; Toi *et al*, 2002; Caine *et al*, 2003; Manders *et al*, 2003; Desruisseau *et al*, 2004; Granato *et al*, 2004; Sancak *et al*, 2004; Zhao *et al*, 2004; Bando *et al*, 2005; O’Riain *et al*, 2005), Supplementary Table S2 for prostate cancer (Joseph *et al*, 1997; Salven *et al*, 1997; Bauer *et al*, 1999; Duque *et al*, 1999; Jones *et al*, 2000; Bok *et al*, 2001; Figg *et al*, 2001; Caine *et al*, 2003, 2004; Kohli *et al*, 2003; George *et al*, 2004; Kaushal *et al*, 2005; Li *et al*, 2005), Supplementary Table S3 for colorectal cancer (Dirix *et al*, 1996; Hyodo *et al*, 1998; Kumar *et al*, 1998; Baker *et al*, 2000; Chin *et al*, 2000; Davies *et al*, 2000; George *et al*, 2000; Broll *et al*, 2001; Haraguchi *et al*, 2002; Karayiannakis *et al*, 2002; Werther *et al*, 2002a, 2002b, 2003) and Supplementary Table S4 for other cancers (Yeo *et al*, 1993; Dosquet *et al*, 1997; Hyodo *et al*, 1998; Tempfer *et al*, 1998; Viac *et al*, 1998; Kraft *et al*, 1999; Salgado *et al*, 1999; Sato *et al*, 1999; Thickett *et al*, 1999; Yanagawa *et al*, 1999; Salven *et al*, 1999a; Matsuyama *et al*, 2000; Stockhammer *et al*, 2000; Yoshikawa *et al*, 2000; Tabone *et al*, 2001; Jacobsen *et al*, 2002; Kishiro *et al*, 2002; Kusumanto *et al*, 2003; Ljungberg *et al*, 2003).

### Cancer studies and meta-analysis

#### Breast cancer

**Figure 1:**
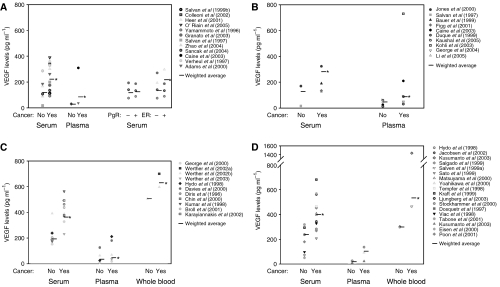
Vascular endothelial growth factor (VEGF) levels in the blood of cancer patients and healthy volunteers. (**A**) Breast cancer studies. (**B**) Prostate cancer studies. (**C**) Colorectal cancer studies. (**D**) Other cancer studies. Weighted average for VEGF values denoted by a bar in the graph. ^*^P<0.001 greater than healthy controls.

**Figure 2:**
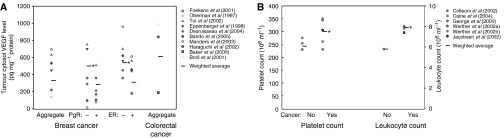
Intratumoural VEGF levels and platelet and leukocyte numbers. (**A**) Tumour cytosol VEGF levels. Colorectal tumour cytosol VEGF values are approximately 1.5 times higher than those in breast cancer studies. (**B**) Platelet count and leukocyte count in cancer studies. ^*^P<0.001 greater than healthy controls.

The main results on healthy and cancer VEGF levels are summarised in Supplementary Table S1 and Figures 1A, 2A and B. Serum VEGF levels in cancer patients appear to be about two times higher than those in healthy controls (range: 92–390 *vs* 17–287 pg ml^−1^). Plasma VEGF levels have a range of 37–310 pg ml^−1^ (cancer) *vs* 27–30 pg ml^−1^ (healthy). Tumour cytosol VEGF values range from 140 to 693 pg mg^−1^ protein, but there is no control equivalent in healthy subjects or other tissues in these studies. We will compare these values with measurements from other tissues reported in other studies.

The relationship between VEGF in cancer and expression of hormone receptors for oestrogen and progesterone was also examined. Serum values appear to be slightly higher for both positive ER and PgR status, although the difference does not appear to be significant (ER status: 75–271 (negative) *vs* 90–298 pg ml^−1^ (positive); PgR status: 75–141 (negative) *vs* 89–187 pg ml^−1^ (positive)). However, both ER and PgR tumour cytosol values deviate from the above data, where ER-negative values are 1.5–2 times higher than ER-positive values (360–700 *vs* 180–560 pg mg^−1^ protein) and PgR-negative values are 1.5 times higher than PgR-positive values (289–760 *vs* 212–510 pg mg^−1^ protein).

#### Prostate cancer

Results are summarised in Supplementary Table S2 and Figures 1B and 2B. Vascular endothelial growth factor level is 2–3 times higher in serum, and 3–10 times higher in plasma of cancer patients (serum: 129–323 cancer *vs* 17–171 pg ml^−1^ (healthy); plasma: 32–730 cancer *vs* 13–61 pg ml^−1^ (healthy)).

#### Colorectal cancer

Results are summarised in Supplementary Table S3 and Figures 1C, 2A and B. Both serum and plasma VEGF are about two times higher in cancer patients (serum: 66–563 cancer *vs* 173–391 pg ml^−1^ (healthy); plasma: 19–211 cancer *vs* 9–126 pg ml^−1^ (healthy)). Whole blood values are relatively high (597–700 cancer *vs* 506 pg ml^−1^ (healthy)), although results may have been skewed with only two studies involved. Tumour cytosol values range from 189 to 984 pg mg^−1^ protein, which are approximately 1.5 times higher than those in breast cancer studies.

#### Other cancer types

Results are summarised in Supplementary Table S4 and Figure 1D. When compared to healthy VEGF, cancer VEGF is elevated by approximately 2–4 times for serum values, and by 2–6 times for plasma values (serum: 207–681 cancer *vs* 51–318 pg ml^−1^ (healthy); plasma: 23–137 cancer *vs* 9–26 pg ml^−1^ (healthy)). For whole blood VEGF, cancer values are again higher when compared to serum and plasma data (461–1435 cancer *vs* 298–301 pg ml^−1^ (healthy)).

#### Summary

Results are summarised in Table 1 and Figure 1A–D. When compared to the ranges of healthy VEGF levels, reported cancer VEGF ranges are about twice as large. The ranges of reported healthy values of VEGF are mostly comparable. Within all groups, the weighted average of normal VEGF is lower than that of the cancer values. Between the groups, however, the average healthy VEGF can be comparable to cancer levels in other groups. For example, average healthy serum VEGF in the other cancer studies is slightly higher than average breast cancer serum VEGF (238 *vs* 222 pg ml^−1^).

### Compartmental analysis and data interpretation

#### Blood compartment

**Figure 3:**
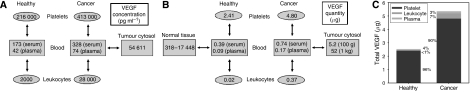
Vascular endothelial growth factor (VEGF) distribution in the body. (**A**) Concentrations of VEGF in the body. Average VEGF concentrations are recorded for platelets, leukocytes and the blood compartment for both healthy and control values. (**B**) Quantities of VEGF in the body. Average VEGF quantities are recorded for platelets, leukocytes and the blood compartment for both healthy and control values. (**C**) Comparison of blood VEGF quantities in healthy and cancer studies. Percentages of VEGF platelets, leukocytes and the blood compartment are evaluated.

To evaluate VEGF distribution in the body, we combined all the studies and derived the average VEGF concentration for serum and plasma. To determine the total quantity of serum or plasma VEGF in the body, we multiplied concentrations by the total volume of serum or plasma, 2.26 l, as explained below (Lentner *et al*, 1984). Based on this analysis, we evaluated the quantity of VEGF in serum to be 4.3 (healthy) and 4.4 (cancer) times higher than the quantity of VEGF in plasma. The results are summarised in Figure 3A and B.

Vascular endothelial growth factor evaluation for platelets is based on two types of measurements: plasma-corrected VEGF/platelet values from Salven *et al* (1999b); George *et al* (2000); and PRP measurements from Kusumanto *et al* (2003) and Salven *et al* (1999a). We excluded reference Caine *et al* (2004) because the data reported was three orders of magnitude higher than those reported in other studies. Concentrations are calculated using volume of a platelet, 9 fl (Lentner *et al*, 1984), and quantity is determined by multiplying the concentration by the total volume of platelets in the blood (cancer: 14.18 ml, healthy: 11.15 ml) (Lentner *et al*, 1984; Werther *et al*, 2002b). Results are summarised in Figure 3A and B. Total quantity of VEGF in platelets is 6.5 times than that in serum, and 28.2 times higher than that in plasma for cancer patients.

Vascular endothelial growth factor is sequestered in the alpha granules of platelets at a high concentration. It has been suggested that platelets might recycle the VEGF they have scavenged, since the VEGF concentration increases in platelets over time for as long as the VEGF source is present (Folkman, 2007). The mechanisms underlying the VEGF sequestration, however, are not well established. Further research is necessary to determine how platelets contribute to the VEGF upregulation in cancer.

Leukocyte VEGF values are determined using PBMNC values from Salven *et al* (1999a). We took the average of granulocyte, lymphocyte and monocytes volumes, 383 fl per cell (Lentner *et al*, 1984). We also calculated the total leukocyte volume in blood (cancer: 13.41 ml, healthy: 9.97 ml) (Lentner *et al*, 1984; Werther *et al*, 2002b). Results are shown in Figure 3A and B. We observe a low quantity of healthy leukocyte VEGF when compared to plasma, serum and platelet levels. However, the difference between healthy and cancer quantities is the highest. The quantity of VEGF in leukocytes in cancer is 18.5 times higher than that in healthy, which is 10 times the increase in plasma, serum or platelet VEGF in cancer.

#### Tumour compartment

We evaluated the average VEGF level in tumour cytosol from the collected data in units of pg mg^−1^ protein. Concentration (pg ml^−1^) was estimated using a protein mass ratio of 160 mg protein g^−1^ fat-free tissue and tissue density of 1.06 g tissue ml^−1^ (Lentner *et al*, 1981). We calculated the total quantity of VEGF based on 100 and 1000 g tumours. Results are summarised in Figure 3A and B.

We observe that the quantity of VEGF in the tumours is 7–70 times higher than the quantity in serum, 31–306 times higher than plasma quantity, 1.1–10.8 times higher than platelet quantity and 14–141 times higher than leukocyte quantity. This indicates that tumour tissue is a significant source and reservoir for VEGF in a cancer patient.

#### Vascular endothelial growth factor content of normal tissues

There is no standard control for tumour cytosol VEGF levels. Vascular endothelial growth factor levels in the normal colon tissues of cancer patients were evaluated (Baker *et al*, 2000; Broll *et al*, 2001). Vascular endothelial growth factor quantities in skeletal muscle (human vastus lateralis) (Gavin *et al*, 2004) and rat muscle, for comparison (Zhang *et al*, 1997) were also evaluated; muscle VEGF content is particularly important since it constitutes close to half the mass of the human body. Vascular endothelial growth factor concentration (pg ml^−1^) in these tissues was estimated using a protein mass ratio of 160 mg protein g^−1^ fat-free tissue and tissue density of 1.06 g tissue ml^−1^ (Lentner *et al*, 1981).

Vascular endothelial growth factor concentration in normal colon tissues and skeletal muscle ranged between 27.3 and 1500 pg mg^−1^ protein or 4630 and 254 400 pg ml^−1^ tissue. The total body mass of VEGF can be evaluated using the above data, the body mass of a 30–39-year-old male (78 kg), the volume of blood (5 l) and the density of blood (1060 kg m^−3^). Assuming that the body consisted only of tissue and blood, we estimated the total VEGF to be between 318 and 17448 *μ*g.

Vascular endothelial growth factor level in other tissues in rats have been reported in units of pg mg^−1^ tissue (Eccles, 2001). Assuming human tissue concentrations similar to these rat concentrations, and using the mass of human organs (Lentner *et al*, 1981), the total quantity of VEGF in each organ was predicted as 1.8 *μ*g in heart, 6.7 *μ*g in liver, 30.6 *μ*g in lungs, 2.4 *μ*g in kidney and 14.6 *μ*g in brain. It thus appears that, of the healthy tissues, skeletal muscle contains the most VEGF.

Compared to the total mass of VEGF in the human body, tumour contributes to a relatively small percentage of VEGF (0.03–2% for 100 g tumour, 0.3–16% for 1 kg tumour). This may indicate that VEGF is more readily compartmentalised in blood and muscle. Further research will be needed to determine how VEGF is concentrated and transported in each of the compartments of the body.

#### Free VEGF concentration in healthy and tumour tissues

The concentration of free (unbound) VEGF in the interstitial space of human breast tumour and muscle vastus lateralis have been measured using microdialysis. For both tissues, this concentration is in the range of 0.5–1.5 pM (Dabrosin *et al*, 2002, 2003; Hoffner *et al*, 2003), or 23–68 pg ml^−1^ interstitial space, or 1.6–4.8 pg ml^−1^ tissue (skeletal muscle), 13.3–39.4 pg ml^−1^ tissue (breast tumour). Note that the extracellular concentration is similar to the plasma concentration (Figure 3A), suggesting that plasma VEGF and interstitial VEGF are close to being at equilibrium as VEGF moves across the endothelial barrier of the vasculature. Our computational models of VEGF transport *in vivo* predict that this free VEGF accounts for approximately 1% of the total extracellular VEGF in the tissue (extracellular VEGF comprises free, extracellular matrix-bound and cell surface receptor-bound) (Mac Gabhann and Popel, 2007). Thus, there is approximately 160–480 pg ml^−1^ tissue extracellular VEGF in the muscle, or 5.1–15.2 *μ*g in total. This suggests that the remaining (i.e., most of VEGF in the body—approximately 8000 *μ*g in skeletal muscle alone, based on concentrations in human vastus lateralis, Gavin *et al*, 2004) is located intracellularly.

#### Other VEGF measurements

Elevated VEGF is also noted in other angiogenic environments. For example, in wound healing, VEGF concentration in the wound fluid is several fold the plasma or serum concentration (Hormbrey *et al*, 2003; Karayiannakis *et al*, 2003; Wu *et al*, 2003; Di Vita *et al*, 2006). In bone marrow fluid, VEGF levels increase from 1.5 to 4 pM in acute leukaemia patients (Ye *et al*, 2003).

Vascular endothelial growth factor levels have also been reported in cancer-associated effusions. Vascular endothelial growth factor concentrations in pleural effusions is about three times higher in cancer patients (of various types of cancer) than in healthy subjects (cancer: 2929 pg ml^−1^, healthy: 930 pg ml^−1^) (Kraft *et al*, 1999; Thickett *et al*, 1999; Yanagawa *et al*, 1999; Matsuyama *et al*, 2000; Kishiro *et al*, 2002). In peritoneal effusions, VEGF values increases from about 20 to 31 pmol l^−1^ in cancer patients (of various types of cancer) (Yeo *et al*, 1993). In these two types of effusions, multiple types of cancer patients are involved (Supplementary Table S3, Online Supplement). In pericardial effusions, lung cancer VEGF levels are about 40-fold the healthy VEGF level (cancer: 3072 pg ml^−1^, control: 81 pg ml^−1^) (Matsuyama *et al*, 2000).

Vascular endothelial growth factor secreted from various tissues will eventually be cleared by the kidney. Urine VEGF values have been reported to be higher in cancer patients when compared to healthy controls (Eisen *et al*, 2000; Bok *et al*, 2001). Renal clearance of VEGF could be responsible for changing VEGF levels in the blood compartment. Future mechanistic studies should delineate whether increased VEGF in plasma of cancer patients is caused by the increased VEGF secretion by tumour and bone marrow cells, or by the altered rates of VEGF clearance from the kidneys.

## CONCLUSION

Our results provide an integrative analysis of VEGF levels in various compartments of the body in cancer patients and address important issues in VEGF data interpretation. Conclusions drawn from VEGF values in different cancer types were consistent. Within the blood compartment, VEGF is mostly concentrated in the platelets, although a significant portion was localised in leukocytes during cancer development. Large quantities of VEGF were reported in tumour and skeletal muscle, the latter of which suggested an intracellular VEGF source.

The most surprising result of this analysis is that even in cancer, tumours are not the largest source of VEGF in the body. Other tissues, and in particular skeletal muscle, appear to contain a large reservoir of VEGF, and this should be a consideration in the design of cancer therapeutics. For example, the systemic administration of anti-VEGF antibodies may have to overcome the effects of a large non-tumour-derived VEGF reservoir.

## Figures and Tables

**Table 1:** Weighted average VEGF of all cancer studies

	***n*** ^*^	**Serum (pg ml^−1^)**	**s.d.**	***n***	**Plasma (pg ml^−1^)**	**s.d.**	***n***	**Whole blood (pg ml^−1^)**	**s.d.**	***n***	**Tumour cytosol (pg** **mg**^−1^ **protein)**	**s.d.**
*Breast cancer*												
Healthy	371 (6)	119	83	75 (2)	28	1	NA	NA	NA	NA	NA	NA
Cancer	883 (11)	222	101	168 (2)	85	105	NA	NA	NA	1797 (7)	329	159
												
*Prostate cancer*												
Healthy	29 (2)	129	70	540 (3)	47	8	NA	NA	NA	NA	NA	NA
Cancer	104 (4)	281	74	771 (6)	87	153	NA	NA	NA	NA	NA	NA
												
*Colorectal cancer*												
Healthy	390 (6)	193	55	147 (4)	35	40	24 (1)	506	NA	115 (2)	62	31
Cancer	931 (9)	363	115	553 (5)	46	36	75 (2)	630	48	119 (3)	612	366
												
*Other cancers*												
Healthy	290 (7)	238	91	33 (2)	19	8	69 (2)	299	1	NA	NA	NA
Cancer	901 (13)	399	137	87 (3)	104	27	56 (2)	533	252	NA	NA	NA
												
*All cancers*												
Healthy	1080 (21)	173	90	825 (12)	42	20	93 (3)	352	91	115 (2)	62	31
Cancer	2833 (37)	328	139	1579 (16)	74	116	131 (4)	589	175	1916 (10)	334	184

^*^Total number of patients (number of papers).

Abbreviation: VEGF=vascular endothelial growth factor.
