# Supplementary material for: Where is VEGF in the body? A meta-analysis of VEGF distribution in cancer
Source: Br J Cancer. 2007 Oct 2;97(7):978–85. doi: 10.1038/sj.bjc.6603923 (PMC2360423; doi:10.1038/sj.bjc.6603923)
Supplement: Supplementary Tables and Information [file 6603923x1.pdf]

Supplement To:

Where is VEGF in the body? A meta-analysis of VEGF distribution in cancer

Carmen Kut, Feilim Mac Gabhann, Aleksander S. Popel

Department of Biomedical Engineering, Johns Hopkins University School of Medicine,

Baltimore, MD 21205

Supplementary Data Tables S1-S6

Full references for each of the citations are given in the main article

SUPPLEMENTARY DATA TABLES:

Table S1. Main Characteristics of the 19 studies involving Breast Cancer

Table S2. Main Characteristics of the 13 studies involving Prostate Cancer

Table S3. Main Characteristics of the 13 studies involving Colorectal Cancer

Table S4. Main Characteristics of the 19 studies involving Other Cancer

Table S5. Main Characteristics of the 6 studies involving Platelet and Leukocyte Count

Table S6. Reason for Exclusion of 12 Studies.

Footnotes for Tables S1-S5

* BC-Breast Cancer, PC-Prostate Cancer, CRC-Colorectal Cancer, RCC-Renal Cell Carcinoma, OC-Ovarian Cancer, GC- Gastric Cancer, GIC - Gastrointestinal Cancer, A - Anal Carcinoma, LC - Lung Cancer, Lym. - Lymphoma, BT - Brain Tumor, HCC – Hepatocellular carcinoma, MT - Malignant Tumor, MTC - Malignant Tumor in Children, M - melanoma

† NA-Not Applicable

‡ ELISA-Enzyme-Linked Immunosorbent Assay, QSEIT – Quantitative Sandwich Enzyme Immunoassay Technique, EIA – Enzyme Immuno Assay, ICMA – Chemiluminescence Immunosorbent Assay, IQK – Human VEGF Immunoassay Quantigo Kit, IFA – Immunofluorometric Assay

§ ben.-Benign, CIS-Carcinoma In Situ, prim.-Primary Cancer, met.-Metastatic Cancer, pre-treat.-Pre-Treatment, all pat.-All Patients, G.(1-3)-Histological Grading, S. (I-IV)-UICC Stage, pre-op.-Pre-operation, rec.-Recurrence, loc.-Localized, dis.-Disseminated, PC – Prostate Cancer, ear.-early, adv.-advanced, HE-Hormone Escaped, BPH-Benign Prostatic Hyperplasia, PSAs – PSA Stable, PSAr – PSA rising, LPF-Localized Prostatic Fluid, pol. – Polyp, DS – Duke’s Stage(A-C), CS-Clinical Stage, rem.-Remission, GIC-Gastrointestinal Cancer, OC-Ovarian Cancer, LC – Lung Cancer, ben. ex. – benign exudative effusion, ben. trans. – benign transdative effusion, indiv. – individual (values reported for each individual patient)

‖ Me-Mean, Md-Median, MQ-Median And Interquartile Range, MR-Median and Range, MCI-Median and 95% Confidence Interval, MSE-Mean and Standard Error, R-Range, SD-Standard Deviation, DPP-Data Per Patient

** ^{e}-Estimate based on reported subgroup data

†† PLT-Platelets, PBMNC-Peripheral Blood Mononuclear Cell

‡‡ number of cancer patients (number of healthy controls)

*** PRP – platelet rich plasma, PPP – platelet poor plasma, PBMNC – peripheral blood mononuclear cell suspension, EPF – expressed prostatic fluid, SVF – seminal vesicular fluid

Table S1. Main Characteristics of the 19 studies involving Breast Cancer

Ref[1]	Year[1]	Cancer[*]	n[‡‡]	VEGF source	VEGF Isoform[†]	VEGF Measurement[‡]	VEGF Diagnosis[§]	Statistics[‖]	healthy VEGF (mean/ *median*)	healthy SD	Cancer VEGF[**] (mean/ *median*)	Cancer SD	unit[††]
(Adams *et al*, 2000)	2000	BC	138 (63)	plasma	NA	ELISA	ben., loc., rem., met.	MR	*27*		*37*[e]		pg/m
(Caine *et al*, 2003)	2003	BC	30 (12)	plasma	165	ELISA	all pat.	MQ	*30*		*310*		pg/m
(Yamamoto *et al*, 1996)	1996	BC	175	serum	121	EIA	S. (I, II,III, rec)	MSE			92[e]		pg/m
(Verheul *et al*, 1997)	1997	BC	27 (30)	serum	NA	QSEIT	pre-treat.	MSE, Me	287		330	206	pg/m
(Salven *et al*, 1997)	1997	BC	33 (113)	serum	165	QSEIT	loc., dis.	MR	*17*		*200*[e]		pg/m
(Salven *et al*, 1999b)	1999	BC	105	serum	NA	QSEIT	ben., CIS, prim, met.	MR			*112*[e]		pg/m
(Adams *et al*, 2000)	2000	BC	138 (63)	serum	NA	ELISA	ben., loc., rem., met.	MR	*186*		*262*[e]		pg/m
(Heer *et al*, 2001)	2001	BC	173 (88)	serum	165	QSEIT	S. (I,II,III,IV)	MQ	*168*		*302*[e]		pg/m
(Colleoni *et al*, 2002)	2002	BC	48	serum	165	ELISA	pre-treat.	MCI			*340*		pg/m
(Granato *et al*, 2004)	2003	BC	51	serum	NA	QSEIT	G. (1-2, 3)	MR			*175*[e]		pg/m
I(Zhao *et al*, 2004)	2004	BC	59 (59)	serum	NA	ELISA	pre-op.	MR	*89*		*365*		pg/m
(Sancak *et al*, 2004)	2004	BC	44 (18)	serum	NA	ELISA	all pat.	MSE	105	49	136	58	pg/m
(O'Riain *et al*, 2005)	2005	BC	30	serum	NA	EIA	pre-op.	MSE			390[e]		pg/m
(Obermair *et al*, 1997)	1997	BC	89	tumor cytosol	165	EIA	G. (1,2,3)	MQ			*140*[e]		pg/m prote
(Eppenberger *et al*, 1998)	1998	BC	305	tumor cytosol	121, 165	ICMA	all pat.	MR			*450*		pg/m prote
(Foekens *et al*, 2001)	2001	BC	845	tumor cytosol	121, 165	ELISA	all pat.	MQ			*220*		pg/m prote
(Toi *et al*, 2002)	2002	BC	110	tumor cytosol	NA	ELISA	all pat.	MSE			630	100	pg/m prote
(Manders *et al*, 2003)	2003	BC	53	tumor cytosol	121, 165	ELISA	G. (1,2,3)	Md			*693*[e]		pg/m prote
(Desruisseau *et al*, 2004)	2004	BC	193	tumor cytosol	121, 165	ELISA	all pat.	MQ, R			*218*		pg/m prote
(Bando *et al*, 2005)	2005	BC	202	tumor cytosol	NA	ELISA	all pat.	MCI			*532*		pg/m prote

Table S2. Main Characteristics of the 13 studies involving Prostate Cancer

Ref[1]	Year[1]	Cancer[*] n[‡‡1]	VEGF source[***]	VEGF Isoform[†]	VEGF Measurement[‡]	VEGF Diagnosis[§]	Statistics[‖]	healthy VEGF (mean/ *median*)	healthy SD	Cancer VEGF[**] (mean/ *median*)	Cancer SD	unit[††]
(Duque *et al*, 1999)	1999	PC 80 (26)	plasma	121,165	QSEIT	loc., met.	MQ, Me	13		*32* [e]		pg/ml
(Caine *et al*, 2003)	2003	PC 30 (12)	plasma	165	ELISA	all pat.	MQ	*27*		*120*		pg/ml
(George *et al*, 2004)	2004	PC 86	plasma	NA	ELISA	pre-op.	MR, MQ			*50*		pg/ml
(Li *et al*, 2005)	2005	PC 504(502)	plasma	NA	ELISA	all pat.	MQ	*49*		*48*		pg/ml
(Kohli *et al*, 2003)	2003	PC 41	Plasma (PPP)	121,165	ELISA	PSAs, PSAr	MR, MQ			*730* [e]		pg/ml
(Kaushal *et al*, 2005)	2005	PC 30	Plasma (P PP)	VEGF-A	ELISA	ear., adv.	MQ			90 [e]		pg/ml
(Salven *et al*, 1997)	1997	PC 8 (113)	serum	165	QSEIT	loc., dis.	MR, MSE	*17*		129 [e]		pg/ml
(Bauer *et al*, 1999)	1999	PC 10	serum	NA	IQK	pre-treat.	MR			*191*		pg/ml
(Jones *et al*, 2000)	2000	PC 78 (21)	serum	NA	ELISA	loc., met., HE, BPH	MSE	171	105	323 [e]		pg/ml
(Figg *et al*, 2001)	2001	PC 8	serum	NA	ELISA	pre-treat.	MSE, Me			136	123	pg/ml
(Caine *et al*, 2004)	2004	PC 30 (30)	PRP	NA	IQK	all pat.	MQ	*600*		*690*		pg/10^6
(Bok *et al*, 2001)	2001	PC 100	urine	NA	ELISA	pre-treat.	MR, MSE			46, *28*	58	pg/ml
(Joseph *et al*, 1997)	1997	PC 48	EPF	NA	ELISA	PC	MSE			1.1x 10^6 [e]		pg/ml
(Joseph *et al*, 1997)	1997	PC 16	SVF	NA	ELISA	LPF	MSE			2.6x 10^4	7x 10^4	pg/ml

Table S3. Main Characteristics of the 13 studies involving Colorectal Cancer

Ref[1]	Year[1]	Cancer[*]	n[‡‡]	VEGF source[***1]	VEGF Isoform[†]	VEGF Measurement[‡]	V EGF Diagnosis[§]	Statistics[‖]	healthy VEGF (mean/ *median*)	healthy SD	Cancer VEGF[**] (mean/ *median*)	Cancer SD	unit[††]
(Hyodo *et al*, 1998)	1998	CRC 39	(20)	plasma	165	ELISA	pol., S. (II-III, IV)	MSE, Md	26	8.2	82 [e]		pg/ml
(George *et al*, 2000)	2000	CRC 116	(84)	plasma	NA	ELISA	all pat.	MQ	*9*		*19*		pg/ml
(Davies *et al*, 2000)	2000	CRC 29	(19)	plasma	121,165	ELISA	all pat.	MQ	*126*		*180*		pg/ml
(Werther *et al*, 2002b)	2002	CRC 51	(24)	plasma	165	ELISA	all pat.	MSE		57	69	83	pg/ml
(Werther *et al*, 2003)	2003	CRC 318		plasma	NA	ELISA	pre-op.	MR			*36*		pg/ml
(Dirix *et al*, 1996)	1996	CRC 44		serum	NA	ELISA	all pat.	MSE,MR			462, *351*	338	pg/ml
(Hyodo *et al*, 1998)	1998	CRC	(20)	serum	165	ELISA	pol., S. (II-III, IV)	MSE, Md	238	125			pg/ml
(Kumar *et al*, 1998)	1998	CRC 108	(136)	serum	121,165	ELISA	S. (I, II, III, IV)	MQ	*174*		*563* [e]		pg/ml
(Chin *et al*, 2000)	2000	CRC 81		serum	121,165	ELISA	S. (I, II-III)	MQ			377 [e]		pg/ml
(George *et al*, 2000)	2000	CRC 116	(84)	serum	NA	ELISA	all pat.	MQ	*152*		*327*		pg/ml
(Broll *et al*, 2001)	2001	CRC 122	(65)	serum	165	ELISA	all pat.	MSE, R	203	124	438	396	pg/ml
(Werther *et al*, 2002a)	2002	CRC 24		serum	NA	ELISA	pre-op.	MSE			375	246	pg/ml
(Werther *et al*, 2002b)	2002	CRC 51	(24)	serum	165	ELISA	all pat.	MSE	391	175	388	249	pg/ml
(Karayiannakis *et al*, 2002)	2002	CRC 67	(61)	serum	165	ELISA	all pat.	MQ	*186*		*492*		pg/ml
(Werther *et al*, 2003)	2003	CRC 318		serum	NA	ELISA	pre-op.	MR			*231*		pg/ml
(George *et al*, 2000)	200	CRC 116	(84)	PCPP	NA	ELISA	pre-op.	MQ	*0.62*		*0.97*		pg/10^6 PLT
(Werther *et al*, 2002b)	2002	CRC 51	(24)	lysed NE	165	ELISA	all pat.	MSE	298	82	382	152	pg/ml
(Werther *et al*, 2002a)	2002	CRC 24		whole blood	NA	ELISA	pre-op.	MSE			700	256	pg/ml
(Werther *et al*, 2002b)	2002	CRC 51	(24)	whole blood	165	ELISA	all pat.	MSE	506	141	597	229	pg/ml
(Baker *et al*, 2000)	2000	CRC 50	(50)	tumor cytosol	NA	ELISA	all pat.	MR	*27*		*189*		pg/mg protein
(Karayiannakis *et al*, 2002)	2001	CRC 38	(65)	tumor cytosol	165	ELISA	all pat.	MSE, R	89	57	984		pg/mg protein
(Haraguchi *et al*, 2002)	2002	CRC 31		tumor cytosol	NA	ELISA	DS (A,B,C)	MSE			840[e]		pg/mg protein

Table S4. Main Characteristics of the 19 studies involving Other Cancer

Ref[1]	Year[1]	Cancer[*]	n[‡‡1]	VEGF source[***1]	VEGF Isoform[†]	VEGF Measurement[‡]	VEGF Diagnosis[§]	Statistics[‖]	healthy VEGF[**] (mean/ *median*)	healthy SD	Cancer VEGF[**] (mean/ *median*)	Cancer SD	unit[††]
(Hyodo *et al*, 1998)	1998	GC 29	(20)	plasma	165	ELISA	S. (I, II-III, IV)	MSE,Md	26	8.2	137 [e]		pg/ml
(Yoshikawa *et al*, 2000)	2000	GC 54		plasma	NA	ELISA	S. (I-II, III-IV)	MSE			93 [e]		pg/ml
(Kusumanto *et al*, 2003)	2003	BC, AC 4	(13)	plasma(PPP)	165	ELISA	all pat.	MR	*9*		*23*		pg/ml
(Dosquet *et al*, 1997)	1997	RCC 76		serum	165	ELISA	loc., met.	MR			*398* [e]		pg/ml
(Hyodo *et al*, 1998)	1998	GC	(20)	serum	165	ELISA	S. (I, II-III, IV)	MSE,Md	238	125			pg/ml
(Tempfer *et al*, 1998)	1998	OC 60		serum	165	ELISA	all pat.	MR			*466*		pg/ml
(Viac *et al*, 1998)	1998	MT 96	(23)	serum	165	ELISA	prim., met.	MR, SD	*97*	89	*217* [e]		pg/ml
(Salgado *et al*, 1999)	1999	LC 80		serum	NA	ELISA	all pat.	MSE, MR			453, *314*	381	pg/ml
(Sato *et al*, 1999)	1999	RCC 40	(40)	serum	NA	QSEIT	all pat.	MSE	72	9	207	33	pg/ml
(Kraft *et al*, 1999)	1999	OC,GIC 83	(145)	serum	165	ELISA	OC, GIC	MR	*294*		*681* [e]		pg/ml
(Eisen *et al*, 2000)	2000	OC, BC, HCC, M 41	(2)	serum	NA	ELISA	pre-treat.	Me, R	51		568		pg/ml
(Matsuyama *et al*, 2000)	2000	LC 49		serum	121,165	ELISA	S.(I,IIA,B, IIIA,B, IV)	MSE			407 [e]		pg/ml
(Stockhammer *et al*, 2000)	2000	BT 14		serum	165	ELISA	all pat.	MR			*283*		pg/ml
(Poon *et al*, 2001)	2001	HCC 100	(20)	Serum	NA	ELISA	all pat.	MR	*180*		*269*		pg/ml
(Tabone *et al*, 2001)	2001	MTC 40	(40)	serum	165	ELISA	all pat.	MR	*318*		*400*		pg/ml
(Jacobsen *et al*, 2002)	2002	RCC 161		serum	165	ELISA	all pat.	MR			*344*		pg/ml
(Ljungberg *et al*, 2003)	2003	RCC 61		serum	165	ELISA	S. (I-II, III, IV)	MR, Me			561 [e]		pg/ml
(Salven *et al*, 1999a)	1999	Lym. 52	(56)	PRP	NA	QSEIT	all pat.	MR	*5*		*10.6*		$pg/10^6$ PLT
(Kusumanto *et al*, 2003)	2003	BC, AC 4	(13)	PRP	165	ELISA	all pat.	MR	*0.48*		*0.7*		$pg/10^6$ PLT
(Salven *et al*, 1999a)	1999	Lym. 52	(56)	PBMNC	NA	QSEIT	all pat.	MR	*0.9*		*10.6*		$pg/10^6$ PBMNC
(Kusumanto *et al*, 2003)	2003	BC, AC 4	(13)	Granulocytes	165	ELISA	all pat.	MR	*72*		*152.5*		$pg/10^6$ cells
(Salven *et al*, 1999a)	1999	Lym. 52	(56)	Whole Blood	NA	QSEIT	all pat.	MR	*298*		*464*		pg/ml
(Kusumanto *et al*, 2003)	2003	BC, AC 4	(13)	Whole Blood	165	ELISA	all pat.	MR	*301*		*1435*		pg/ml
(Stockhammer *et al*, 2000)	2000	BT 14		cyst fluid	165	ELISA	all pat.	MR, Me			31600		pg/ml

(Eisen *et al*, 2000)	2000	OC, BC, HCC, M	41	(2)	urine	NA	ELISA	pre-treat.	Me, R	83		106		pg/ml
(Kraft *et al*, 1999)	1999	OC	35		pleural effusion	165	ELISA	all pat.	MR			*5528*		pg/ml
(Kraft *et al*, 1999)	1999	GIC	48		pleural effusion	165	ELISA	all pat.	MR			*813*		pg/ml
(Thickett *et al*, 1999)	1999	MT	40	(40)	pleural effusion	NA	ELISA	all pat.	Md	*305*		*2500*		pg/ml
(Yanagawa *et al*, 1999)	1999	LC	111		pleural effusion	165	EIA	LC, ben. ex,, ben. trans	MSE	1567[e]	897	2920	820	pg/ml
(Matsuyama *et al*, 2000)	2000	LC	6		pleural effusion	121, 165	ELISA	all pat.	MSE			532	285	pg/ml
(Kishiro *et al*, 2002)	2002	LC	5	(2)	pleural effusion	121, 165, 189	ELISA	all pat.	MSE	666	259	17526	22398	pg/ml
(Yeo *et al*, 1993)	1993	MC	7		peritoneal effusion	NA`	IFA	all pat.	DPP	21		30		pmol/L
(Matsuyama *et al*, 2000)	2000	LC	3		pericardial effusion	121, 165	ELISA	all pat.	MSE			3072	81	pg/ml

Table S5. Main Characteristics of the 6 studies involving Platelet and Leukocyte Count

Ref[1]	Year[1]	Cancer[*]	n[‡‡1]	Diagnosis[§]	Statistics[‖]	Healthy Platelet # (mean/ *median*)	Healthy PLT SD	Cancer Platelet # (mean /*median)*	Cancer PLT SD	Healthy Leukocyte # (mean/ *median)*	Healthy LK SD	Cancer Leukocyte # mean/ *median*	Cancer LK SD	unit[††]
(George *et al*, 2000)	2000	CRC	116 (84)	all pat.	Me	230								10^6/ml
(Colleoni *et al*, 2002)	2002	BC	48	pre-treat.	MCI			*230.5*						10^6/ml
(Werther *et al*, 2002a)	2002	CRC	24	pre-treat.	MSE			345	111			8		10^6/ml
(Werther *et al*, 2002b)	2002	CRC	51 (24)	all pat.	MSE	276	83	351	121	5.8	1.5	7.8	1.9	10^6/ml
(Jacobsen *et al*, 2002)	2002	RCC	161	all pat.	MR			*306*				*7.4*		10^6/ml
(Caine *et al*, 2004)	2004	PC	30 (30)	all pat.	MSE	254,000	36,000	261,000	60,000					10^6/ml

Table S6. Reason for Exclusion of 12 Studies.

Author	Reason of Exclusion
(Bhujwalla *et al*, 2001)	in vitro study
(Huss *et al*, 2001)	no quantitative data available
(Singh *et al*, 2004)	in vitro study
(Mabjeesh *et al*, 2003)	in vitro study
(Kelavkar *et al*, 2001)	in vitro study
(Liu *et al*, 1999)	in vitro study
(George *et al*, 2001)	does not provide mean/median values
(Calvo *et al*, 2002)	in vitro study
(Santini *et al*, 2002)	aberrant data
(May *et al*, 2005)	in vitro study
(Feldman *et al*, 2001)	aberrant data
(Cianchi *et al*, 2004)	in vitro study
(Haggstrom *et al*, 2000)	in vitro study
